# Endogenous health risks, poverty traps, and the roles of health insurance in poverty alleviation

**DOI:** 10.1186/s13561-022-00370-2

**Published:** 2022-04-19

**Authors:** Pu Liao, Xun Zhang, Wanlu Zhang

**Affiliations:** 1 China Institute for Actuarial Science/School of Insurance, Central University of Finance and Economics, 100081 Beijing, China; 2 School of Insurance, Central University of Finance and Economics, Beijing, 100081 China

**Keywords:** Physical overdraft, Health risks, Human capital, Economic development, Poverty

## Abstract

**Background:**

Family education investment is a key factor in reducing intergenerational transmission of poverty. At the price of higher health risk, the poor may overdraw their bodies to earn more money to invest in education. This study investigates the effect of physical overdraft, health risks and health insurance on poverty and economic growth.

**Methods:**

This paper proposes an economic development model of endogenous health risks and poverty by setting up a physical overdraft decision. Furthermore, we introduce mutual health insurance mechanism to analyze its poverty alleviation effects.

**Results:**

First, this study shows that health risks weaken the economy and are among the leading causes of poverty. Second, mutual health insurance can alleviate, but not completely eliminate, the negative impact of health risks on the economy. Third, appropriate health insurance arrangements can lift some or even all poor households out of poverty.

**Conclusion:**

Health risks have a significant effect on poverty. Furthermore, health insurance mechanisms have the advantages of transferring health risks, reducing poverty and improving health equity.

## Background

Poverty remains the worst problem in the world and anti-poverty is one of the key concerns of economists and policymakers. In 2021, it was estimated that 711 million people (i.e., about 10% of the global population) still living in extreme poverty-defined with less than $1.90 per day [30]. Even in the United States, the most developed country in the world, 37 million people were living in poverty in 2020,^1^ which represents 11% of the national population.

Many scholars have studied the causes of poverty and found the intergenerational transmission of poverty [13, 42]. That is, poverty is transmitted from one generation to another, with poor parents having poor children, who are more likely to become poor adults themselves. However, the intergenerational transmission of poverty is affected by many factors, including family composition, parental education, parental health, productive assets, family education investment, domestic violence and other family factors, as well as non-family factors such as social network, conflict, cultural and psycho-social factors, class and caste, religion and ethnicity [10].

Based on the family economic resource model,^2^ this paper studies the intergenerational transmission of poverty and poverty alleviation, and only considers the relevant factors of normal families,^3^ including family assets, education investment and parental health. Among these factors, parental education investment is assumed to be the most significant factor to affect the intergenerational transmission of poverty [8, 9, 23]. Education develops cognitive abilities and skills that make workers more productive and richer than before and improves their socio-economic status [1, 40]. Therefore, parental investment in their children’s education (including time and money), particularly early education, is very important to improve their children’s income in the future [17, 45, 46]. However, children from poor families are much less likely to attend school and have difficulty obtaining high-quality education compared to those from wealthier families [8], and then are more likely to fall into poverty [38].

Family education investment and intergenerational transmission of economic status (poverty) have been widely and deeply studied. Becker and Tomes (1979, 1986) are pioneering articles in this area [5, 6]. They equate human capital with an investment and study family’s investment in education and its impact on the intergenerational transmission of lifetime income and wealth in a two-period equilibrium model. Loury (1981) studies the interaction between the distribution of incomes and intergenerational transfers by assuming that parents cannot borrow to make human capital investments in their offspring, and that the random assignment of abilities to individuals by nature [34]. Glomm and Ravikumar (1992) develop an OLG model with heterogeneous agents to study the distinction between economies with public education and those with private education, and find that income inequality declines more quickly under public education [19]. Galor and Ziera (1993) analyze the role of wealth distribution in macroeconomics through investment in human capital and show that there are multiple steady states in an economy where credit market is imperfect and investment in human capital is indivisible [18]. Restuccia and Urrutia (2004) provide a quantitative model to analyze the impacts of innate ability, early education, and college education on intergenerational human capital transmission, they find that approximately one-half of the intergenerational correlation in earnings is explained by parental investment in education, particularly early education [39]. Lee and Seshadri (2019) develop a multi-period OLG model to analyze the impact of parental and individual education investment on the intergenerational transmission of economic status, and find that (early) education subsidies significantly reduce the intergenerational persistence of poverty [31]. Caucutt and Lochner (2020) develop an intergenerational model of lifecycle human capital accumulation to study the role of early and late investments in children when credit markets are imperfect, their results show that early interventions in education tend to be more successful than later interventions in education in improving human capital outcomes [12].

However, the literature above does not consider parental health risks when studying family education investment and intergenerational transfer of poverty. Parental health status is another key factor in affecting intergenerational transmission of poverty [10]. Parental good health is a key asset and health shocks have been identified as a key driver of downward mobility due to the lost labor income and the costs of seeking treatment [24]. Parental poor health has strong and long-lasting effects on the economic well-being of children in families experiencing downward socioeconomic mobility and increases their exposure to poverty [48]. Due to potential health risks and lack of health insurance system, the poor may change economic behaviors, such as reducing investment in children’s education [14]. This paper contributes to the area of family education investment and the intergenerational transmission of poverty by considering parental health risks, that is, parents may lose their labor income and have to pay medical expenses due to a health shock in the life cycle.

Moreover, all the studies mentioned above assume that households’ income is determined once their human capital is determined and households cannot earn more money in any way. But in fact, households could make more money by working overtime and engaging in dangerous work [20, 26]. Meanwhile, working overtime often increases health risks [11, 16, 44, 47]. Another contribution of this study is that it allows parents to earn more money at the price of a physical overdraft and higher health risk.

This study is motivated by the following questions. Given that investments in education reduce the intergenerational transmission of poverty [12, 31, 39], will parents overdraw their bodies to get more income to invest in children’s education to help them escape the poverty trap? If physical overdraft means higher health risks in the future, how will parents’ decisions on physical overdraft and education investment change? Furthermore, how will physical overdraft affect the intergenerational transmission of poverty? As a tool to manage health risks, what is the role of health insurance arrangements in poverty alleviation?^4^

We have three major assumptions in this study. Firstly, heterogeneous households have different human capitals, which are determined by their parents’ human capitals and education investment. This assumption is the same as in many literature, such as [12, 19, 31, 39].

Secondly, heterogeneous households own the same physical endowment. That is, although the cognitive abilities and skills may be different, each household owns a healthy body in adulthood. Lack of food, clean water and malnutrition may cause the poor to die early or become weak and ill in adulthood [24]. Such households and their offspring may be destined to continue to struggle in the poverty trap. But in general, most households will survive with a healthy body in their 20s. Consequently, this paper takes healthy households as the research object.

Thirdly, and most importantly, households can overdraw their bodies to obtain more income, but the more serious the physical overdraft are, the greater the health risk in the future. Overdraft mentioned in this paper includes working overtime, hard work, poor working environment, engaging in dangerous work, and so on. Households could earn more money by overdrawing their bodies [20, 26]. However, physical overdraft and health risk are strongly correlated. Due to the low cognitive abilities and skills, the poor are likely to engage in informal and precarious employments in order to sustain their necessities [3, 26]. Informality usually means physical overdraft because of its unregulated and unregistered characteristics [35], and is highly correlated with poor health [2, 41]. Even if informal employment is not involved, working overtime often increases health risks [11, 16, 44, 47].

Following [12, 31], this paper develops a four-period overlapping generation model with the above three assumptions. In our model, households in their childhood make no decisions and are raised/educated by their parent; young households bear children and make decisions of physical overdraft, investments in their children’s human capital and savings; households are exposed to health risks in middle age and make consumption and savings decisions; and old households consume all their savings and the remaining physical endowments and die.

Following the same numerical method as [31], this paper analyzes the impacts of health risk on education investment, intergenerational transmission of poverty and economic growth in equilibrium, and discusses the roles of health insurance in poverty alleviation. Our results show that all households will escape the poverty trap in an economy where physical overdraft increases income without risk to health. However, in an economy where health risk is correlated with physical overdraft, physical overdraft will increase health risk and change households’ behaviors, so some households will fall into the poverty trap. In this case, health insurance arrangement can lift some or even all poor households out of poverty by alleviating the negative impact of health risk on the economy.

The remainder of this paper is organized as follows. Section 2 presents our model, and Section 3 calibrates our model parameters and show our main results. This includes parental overdraft decisions with and without health risk and the poverty in the steady states, as well as the role of health insurance in anti-poverty and economic growth. Section 4 concludes.

## Methods

In this study, an OLG model with physical overdraft decisions is developed to describe the economy. The hypothetical initial economy has a large number of heterogeneous households with different levels of human capital *h*_*i*_, where *h*_*i*_ ∈ (0, 1]. Each household’s lifetime is divided into four periods: childhood (0–19 years old), youth (20–39 years old), middle age (40–59 years old), and old age (60–80 years old). Thus, there are four generations in the economy at any time. Assuming that the number of households in each generation is *N*, so the total number of people in the economy at any time is 4*N*.

### Households

#### Childhood

Nurtured by parents, households in their childhood do not make any decisions and their human capital $\hat{h_i}$ is determined by their parents’ human capital *h*_*i*_ and investment in education *e*_*i*_ [12, 19, 31, 39]. We assume that the relationship between $\hat{h_i}$ and *h*_*i*_ can be expressed as follows^5^:

1 $$ \hat{h_i}=f\left({h}_i,{e}_i\right)={h_i}^{\frac{E}{E+{e}_i}}, $$

where $$ {f}_{e_i}^{\prime}\left({h}_i,{e}_i\right)>0 $$, $$ {f}_{h_i}^{\prime}\left({h}_i,{e}_i\right)>0 $$, *f*(*h*_*i*_, 0) = *h*_*i*_, $$ \underset{e_i\to \infty }{\lim }f\left({h}_i,{e}_i\right)=1 $$.

#### Youth

Households in their youth have a human capital endowment and a physical endowment. The former (*h*_*i*_) is determined by their parents, i.e., eq. (1). In addition, each household is born with the same physical endowment, which is standardized in this study by setting *d*_1_ = 1. Households make physical overdraft decision *a*_*i*2_ (0 ≤ *a*_*i*2_ ≤ 1) to compensate for deficiencies in the human capital endowment, and then combine their human capital with the physical overdraft to supply the labor market and earn wages. The combined labor supply of the youth household *l*_*i*2_ is:

2 $$ {l}_{i2}\left({h}_i,{a}_{i2}\right)=\frac{h_i+{a}_{i2}^{\delta }}{1+{a}_{i2}^{\delta }}, $$

where *δ* denotes the effectiveness of physical overdraft. Obviously, $\frac{\partial {l}_{i2}}{\partial {a}_{i2}}>0$, and the sign of $\frac{\partial^2{l}_{i2}}{\partial {a_{i2}}^2}$ depends on the value of *δ*. The range of *l*_*i*2_ is $\left[{h}_i,\frac{1+{h}_i}{2}\right]$.

Assuming that the unit wage of the combined labor force in period *t* is *w*_*t*_, the household’s total income in that period is *w*_*t*_*l*_*i*2_. Household will raise a child in that period and spend their income on consumption, savings, and education. That is:

3 $$ {w}_t{l}_{i2}={c}_{i2}+{s}_{i2}+{e}_i $$

Suppose households subject to a borrowing constraint, i.e., *s*_*i*2_ ≥ 0. Following Kovacevic and Pflug (2011), we further assume that households also subject to a consumption threshold; that is, when the total income is less than the consumption $\underset{\_}{c}$, households have to spend all their income on consumption [29]. Therefore, *s*_*i*2_ = 0, *e*_*i*_ = 0 and *c*_*i*2_ = *w*_*t*_*l*_*i*2_ if ${w}_t{l}_{i2}<\underset{\_}{c}$.

The utility of youth household *V*_2_ is derived from current consumption *u*(*c*_*i*2_), their future expected utility *V*_3_, and the lifetime utility of their offspring ${\hat{V}}_2$. Thus, the lifetime utility maximization problem for the youth household can be derived as follows:

4 $$ {V}_2\left({h}_i\right)=\underset{s_{i2},{e}_i,{a}_{2i}}{\max }u\left({c}_{i2}\right)+\beta E\left({V}_3\left({A}_{i3},{d}_{i2},{H}_i\right)\right)+\beta \varphi {\hat{V}}_2\left(\hat{h_i}\right), $$

where *β* denotes the utility discount factor. *A*_*i*3_ denotes the assets owned by the household at the start of middle age, which are the sum of principal and interest of youth savings, i.e., *A*_*i*3_ = *s*_*i*2_(1 + *r*_*t* + 1_). *d*_*i*2_ is household’s physical state at the start of middle age, i.e., *d*_*i*2_ = 1 − *a*_*i*2_. *H* denotes the household’s health state during the middle age, and *H* ∈ {*healthy*,  *sick*}. The probability of contracting a disease during the middle age depends on the physical state, that is, *p*_*i*_(*H* = *sick*) = 1 − *p*_*i*_(*H* = *healthy*) = 1 − *p*(*d*_*i*2_), where *p*(∙) ∈ [0, 1] and *p*^′^(*d*_*i*2_) > 0. *φ* denotes the extent of altruistic motivation, that is, the extent to which households prefer the lifetime utility of their children.

#### Middle age

In the middle age, household’s children become independent and self-sufficient. Households in their middle age make midlife decisions based on the assets and physical state generated by decisions made in youth. They decide the physical overdraft *a*_*i*3_ based on their human capital *h*_*i*_ to provide the combined labor *l*_*i*3_, that is:

5 $$ {l}_{i3}\left({h}_i,{a}_{i3}\right)=\frac{h_i\left(1+\theta \right)+{a}_{i3}^{\delta }}{1+{a}_{i3}^{\delta }}, $$

where *θ* denotes the growth rate of human capital based on their experiences and skills.

Household exposes to health risk in the middle age. The probability of illness depends on their physical overdraft *a*_*i*2_ in youth and the physical state *d*_*i*2_ (i.e., *p* = 1 − *p*(*d*_*i*2_)). The costs of illness on households include reduction in labor supply, decrease in productivity and increase in medical costs. We monetize all effects by assuming that households pay additional costs *m* when they become ill. Therefore, healthy households (*H*_*i*_ = *healthy*) have a budget constraint of

6 $$ {A}_{i3}+{w}_{t+1}{l}_{i3}={c}_{i3}+{s}_{i3}, $$

and sick households (*H*_*i*_ = *sick*) have a budget constraint of

7 $$ {A}_{i3}+{w}_{t+1}{l}_{i3}={c}_{i3}+{s}_{i3}+m. $$

Meanwhile, they all subject to a borrowing constraint (*s*_*i*2_ ≥ 0) and a consumption threshold $\underset{\_}{c}$; that is, if ${A}_{i3}+{w}_{t+1}{l}_{i3}-m{1}_{\left\{{H}_i= sick\right\}}<\underset{\_}{c}$, then *s*_*i*3_ = 0 and ${c}_{i3}={A}_{i3}+{w}_{t+1}{l}_{i3}-m{1}_{\left\{{H}_i= sick\right\}}$.

The utility of middle-aged households *V*_3_ comes from current consumption *u*(*c*_*i*3_) and their future expected utility *V*_4_. The lifetime utility maximization problem is expressed as:

8 $$ {V}_3\left({A}_{i3},{d}_{i2},{H}_i,{h}_i\right)=\underset{s_{i3},{a}_{i3}}{\max }u\left({c}_{i3}\right)+\beta {V}_4\left({A}_{i4},{d}_{i3}\right), $$

where *A*_*i*4_ denotes the assets owned by households before old age, that is, the sum of principal and interest on middle-age savings *A*_*i*4_ = *s*_*i*3_(1 + *r*_*t* + 2_). *d*_*i*3_ denotes the physical condition of households before old age, that is, *d*_*i*3_ = *d*_*i*2_ − *a*_*i*3_.

#### Old age

During this period, households make the consume decision based on their savings from the middle age and their current physical endowments. Given that households die at the end of this period, we monetize all their physical endowments for consumption, so the lifetime utility maximization problem is:

9 $$ {V}_4\left({A}_{i4},{d}_{i3}\right)=u\left({c}_{i4}\right), $$

where *c*_*i*4_ = *A*_*i*4_ + *ρd*_*i*3_ and *ρ* is the monetary value of the physical endowments *d*_*i*3_.^6^

### Firms

Suppose the economy has identical firms that produce a homogeneous good (*Y*) by combining capital (*K*) and labor (*L*) through a Cobb-Douglas production function with constant returns to scale:

10 $$ Y=F\left(K,L\right)=A{K}^{\alpha }{L}^{1-\alpha }, $$

where parameter *A* > 0 measures the technological output, and 0 < *α* < 1 is the capital share. Firms determine the amount of capital and labor hired based on the price of capital and labor. Thus, their optimization problem is:

11 $$ \underset{K,L}{\max }\ A{K}^{\alpha }{L}^{1-\alpha }-\left(1+r\right)K- wL. $$

Then, we define $k=\frac{K}{L}$, so the solution of the optimization problem above can be derived as follows:

12 $$ \alpha A{k}^{\alpha -1}=1+r, $$

13 $$ \left(1-\alpha \right)A{k}^{\alpha }=w. $$

### The baseline equilibrium

We define *G*_*t*_ as the distribution of human capital of the *t*-th generation of youth households, then ${\int}_0^1d{G}_t=1$. Thus, the total labor supply during the *t*-th period is:

14 $$ {L}_t^s=N{\int}_0^1{l}_{i3}^{\left(t-1\right)}d{G}_{t-1}+N{\int}_0^1{l}_{i2}^{(t)}d{G}_t, $$

where ${l}_{i3}^{\left(t-1\right)}$ denotes the optimal labor supply at time *t* of the *t* − 1 ^th^ generation households during the middle age with a human capital *i*. Similarly, ${l}_{i2}^{(t)}$ denotes the optimal labor supply at time *t* of the *t* ^th^ generation households during the youth with an initial human capital *i*. The total supply of capital is

15 $$ {K}_t^s=\mathrm{N}{\int}_0^1{s}_{i3}^{\left(t-2\right)}d{G}_{t-2}+N{\int}_0^1{s}_{i2}^{\left(t-1\right)}d{G}_{t-1}, $$

where ${s}_{i3}^{\left(t-2\right)}$ denotes the optimal savings at time *t* of the *t* − 2 generation households during the middle age with an initial human capital *i*. Similarly, ${s}_{i2}^{\left(t-1\right)}$ denotes the optimal savings at time *t* of the *t* − 1 generation residents during the youth with an initial human capital *i*.

**Definition:** The economy reaches a stable equilibrium if prices (*w*, *r*) and decision rules satisfy the following conditions:

- Given prices, households of all ages make optimal choice;
- The representative firm maximizes its profit;
- Market clearing and stable equilibrium:

16 $$ {K}_t={K}_t^s=\overline{K},{L}_t={L}_t^s=\overline{L}. $$

- The distribution of human capital is stable (i.e., ${G}_t={G}_{t+1}=\overline{G}$).

### Equilibrium with health insurance

Is there a health insurance arrangement that can help the poor escape poverty? Answering this question is very significant because it directly affects households’ decision. We consider a mutual health insurance system in which households pay a percentage *τ* of their wages during their youth when they have no health risks. The system compensates them when they experience health risks at middle age. Two insurance plans are assumed in this system:

- Plan A: All households pay a health insurance tax.
  - Plan B: Only the households above the consumption threshold pay a health insurance tax.

#### Plan A and the equilibrium with plan A

The budget constraint for youth households with plan A becomes:

17 $$ \left(1-\tau \right){w}_t{l}_{i2}={c}_{i2}+{s}_{i2}+{e}_i. $$

Similarly, households subject to a borrowing constraint (*s*_*i*2_ ≥ 0) and a consumption threshold $\underset{\_}{c}$, that is, if $\left(1-\tau \right){w}_t{l}_{i2}<\underset{\_}{c}$, then *s*_*i*2_ = 0, *e*_*i*_ = 0 and *c*_*i*2_ = (1 − *τ*)*w*_*t*_*l*_*i*2_. In this system, the medical cost is covered when the middle-aged household suffers a health shock. Then, regardless the household is healthy or unhealthy, the budget constraint of the middle-aged household is the same as eq. (6), i.e.,

18 $$ {A}_{i3}+{w}_{t+1}{l}_{i3}={c}_{i3}+{s}_{i3}. $$

We further assume that the system is self-financing because we develop a closed economy. Accordingly, the total tax revenue of all youth households is equal to the total healthcare cost during their middle age. Therefore, *τ* is determined by the following equation:

19 $$ \left(1+{r}_{t+1}\right)N{\int}_0^1\tau {w}_t{l}_{i2}d{G}_t= Nm{\int}_0^1p\left({d}_{i2}\right)d{G}_t, $$

and the total supply of capital based on plan A is:

20 $$ {K}_t^s=N{\int}_0^1{s}_{i3}^{\left(t-2\right)}d{G}_{t-2}+N{\int}_0^1{s}_{i2}^{\left(t-1\right)}d{G}_{t-1}+N{\int}_0^1\tau {w}_{t-1}{l}_{i2}^{\left(t-1\right)}d{G}_{t-1} $$

Compared with Eq. (15), Eq. (20) includes the capital accumulated by the health insurance system $N{\int}_0^1\tau {w}_{t-1}{l}_{i2}^{\left(t-1\right)}d{G}_{t-1}$.

#### Plan B and the equilibrium with plan B

The budget constraint for youth households with plan B becomes:

21 $$ \left(1-\tau \bullet {1}_{\left\{\left(1-\tau \right){w}_t{l}_{i2}\ge \underset{\_}{c}\right\}}\right){w}_t{l}_{i2}={c}_{i2}+{s}_{i2}+{e}_i, $$

where 1_{∙}_ is the indicator function, that is, if $\left(1-\tau \right){w}_t{l}_{i2}\ge \underset{\_}{c},$ then 1_{∙}_ = 1 and zero otherwise. Similarly, households subject to a borrowing constraint (*s*_*i*2_ ≥ 0) and a consumption threshold $\underset{\_}{c}$. The budget constraint remains unchanged as shown in Eq. (18) regardless the middle-aged household is healthy or unhealthy. We also assume the system is self-financing. That is, the total tax revenue of households during the youth is equal to the total healthcare cost during their middle age. Therefore, *τ* is determined by the following equation:

22 $$ \left(1+{r}_{t+1}\right)N{\int}_0^1\tau {w}_t{l}_{i2}\bullet {1}_{\left\{\left(1-\tau \right){w}_t{l}_{i2}\ge \underset{\_}{c}\right\}}d{G}_t= Nm{\int}_0^1p\left({d}_{i2}\right)d{G}_t. $$

and the total supply of capital based on plan B is:

23 $$ {K}_t^s=N{\int}_0^1{s}_{i3}^{\left(t-2\right)}d{G}_{t-2}+N{\int}_0^1{s}_{i2}^{\left(t-1\right)}d{G}_{t-1}+N{\int}_0^1\tau {w}_{t-1}{l}_{i2}^{\left(t-1\right)}\bullet {1}_{\left\{\left(1-\tau \right){w}_t{l}_{i2}\ge \underset{\_}{c}\right\}}d{G}_{t-1}. $$

## Results

It is difficult to obtain closed-form solutions for models above. Therefore, we set the parameters based on the literature and the actual situation in China to solve the model numerically and analyze it quantitatively.

### Calibration

#### Preference

Following the assumption of İmrohoroğlu and Zhao (2018), we assume the utility function is a constant relative risk aversion function, and the relative risk-aversion coefficient is set at *γ* = 3 [27]. Referring to [32, 49], the one-year utility discount factor is set at 0.98 (i.e., *β* = 0.98^20^). The extent of the altruistic motivation *φ*, which measures the extent of parental preference for the lifetime utility of the offspring, is set at 1 in this study.

#### Human capital and labor

Parameter *E* controls the effectiveness of educational inputs in improving next generation’s human capital. The larger the *E*, the less effective the educational input. In this study, we assume *E* = 50,000 yuan based on the range of wages. *δ* controls the effectiveness of physical overdraft to boost the human capital. Given the fact that physical overdraft *a* ∈ (0, 1), the larger the *δ*, the less effective the physical overdraft. We set *δ* = 0.7. *θ* measures the increment in the human capital from work experience, which is assumed to be *θ* = 0.5. In this study, we set *ρ* = 500,000 yuan based on the range of wages, which measures the psychological price of the physical state (i.e., the expenses that households in old age need before they die).

#### Production function

*α* measures the output elasticity of capital. Referring to [49], this study sets *α* = 0.35. *A* measures total factor productivity, commonly used in numerical simulations to regulate wages and interest rates [31], and is set at *A* = 20,000 in the paper.

#### Living standards and medical costs

Households subject to a consumption threshold; that is, households spend all their income on consumption if their income is lower than consumption. According to the China Ministry of Civil Affairs’ statistics for the fourth quarter of 2020,^7^ the average urban (rural) minimum subsistence level is 8131.2 (5962.3) yuan per person per year. Thus, we assume $\underset{\_}{c}=\mathrm{7,000}\frac{\mathrm{yuan}}{\mathrm{person}}\times 20\ \mathrm{years}=\mathrm{140,000}$ yuan. According to the China Family Panel Studies in 2016 (CFPS 2016), the average medical expenses per person over the past 12 months is 19,182.25 yuan; thus, we assume $m=\mathrm{20,000}\frac{\mathrm{yuan}}{\mathrm{person}}\times 20\ \mathrm{years}=\mathrm{400,000}$ yuan.

#### Population

We set the population size for each generation at *N*. The distribution of human capital for each generation is assumed to be the same at the beginning, so *G*_0_ = *G*_−1_ = *G*_−2_. According to [32], the income distribution of Chinese rural residents can be fitted with a log-logistic distribution with parameters 8.8404 and 0.4342. Therefore, this study approximates log-logistic (8.8404, 0.4342) as the initial human capital distribution.

### Results without health insurance

#### Dynamic paths without health insurance

**Fig. 1:**
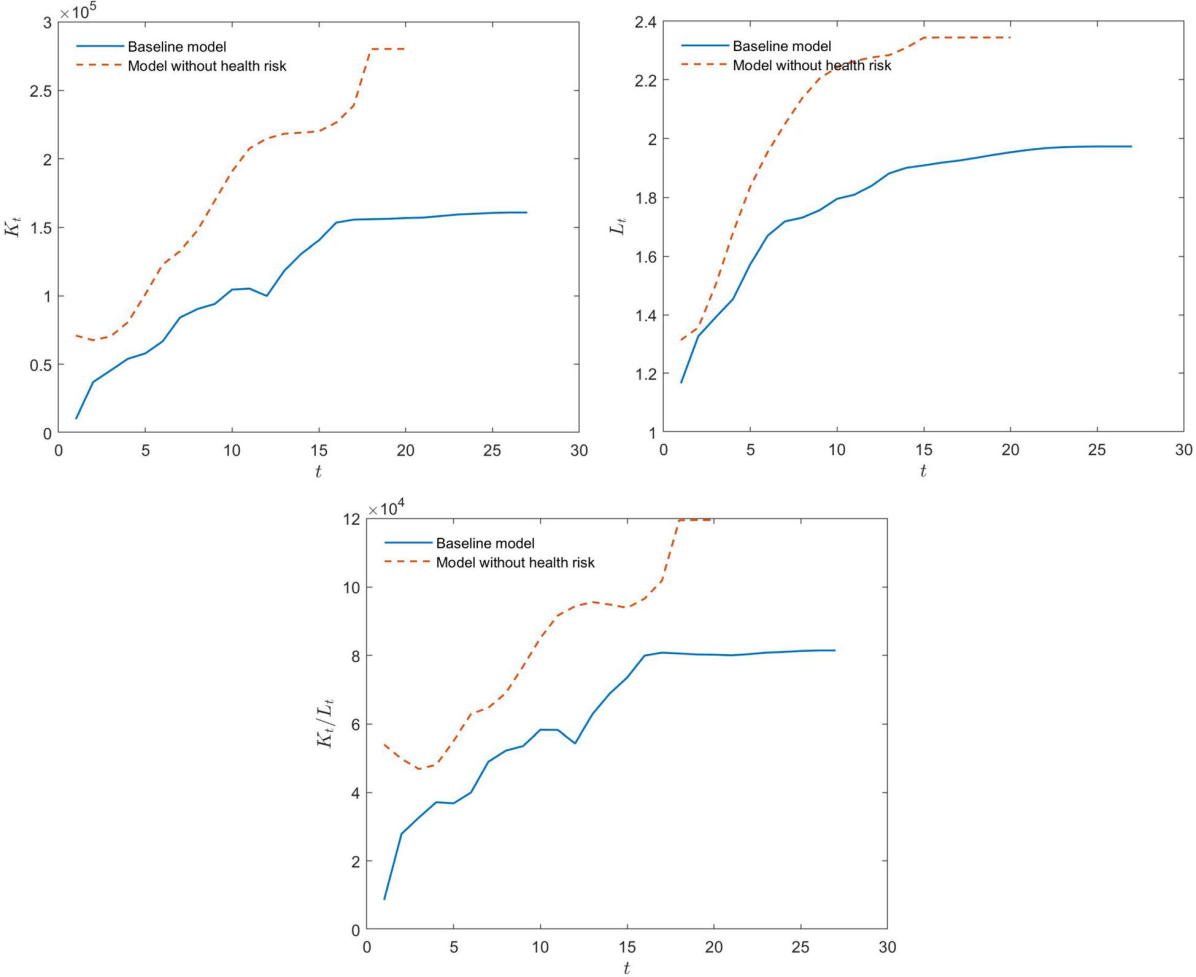
Dynamic paths for the baseline and no-health-risk models (The initial wage is calculated according to the per capita disposable wage income of urban residents in China in 2020, which was 26,381 yuan; thus, *w*_0_ = 520,000. The initial interest rate is calculated according to the annualized rate of return of 3%; thus, *r*_0_ = 0.8061)

Based on the parameters in Table 1, we can solve households’ utility maximization problems and determine the dynamic paths of total labor, total capital, and capital per capita (= total capital/total labor), which are shown in Fig. 1.

**Table 1:** Benchmark parameters values

Symbol	Parameter	Calibration
*γ*	Risk aversion factor	3
*β*	Utility discount factor	0.98^20^
*φ*	Altruistic motivation	1
*E*	Effectiveness of educational inputs	50,000 yuan
*δ*	Effectiveness of physical overdraft	0.7
*θ*	Growth rate of human capital	0.5
*ρ*	Psychological price of physical state	50,000 yuan
*α*	Capital share	0.35
*A*	Total factor productivity	20,000
$\underset{\_}{c}$	Consumption threshold	140,000 yuan
*m*	Medical expenses	400,000 yuan
*G*_0_, *G*_−1_, *G*_−2_	Distribution of initial human capital levels	log-logistic(8.8404, 0.4342)

The results of the baseline model in Fig. 1 are the dynamic paths of solutions for Eqs. (1)–(16) based on the parameters in Table 1. The results of the model without health risks are the dynamic paths of solutions for Eqs. (1)–(16) based on the parameters in Table 1 with the assumption of medical expenses *m* = 0 instead of m = 400,000. To highlight the effect of health risks on the economy, we compare the dynamic paths of these two models. According to Fig. 1, the total capital, the total labor, and the capital per capita with health risks in the steady state are lower than that without health risks. Therefore, health risks have an adverse effect on the economy. We explain this result in the next subsection.

#### Steady states and the poverty trap without health insurance

**Fig. 2:**
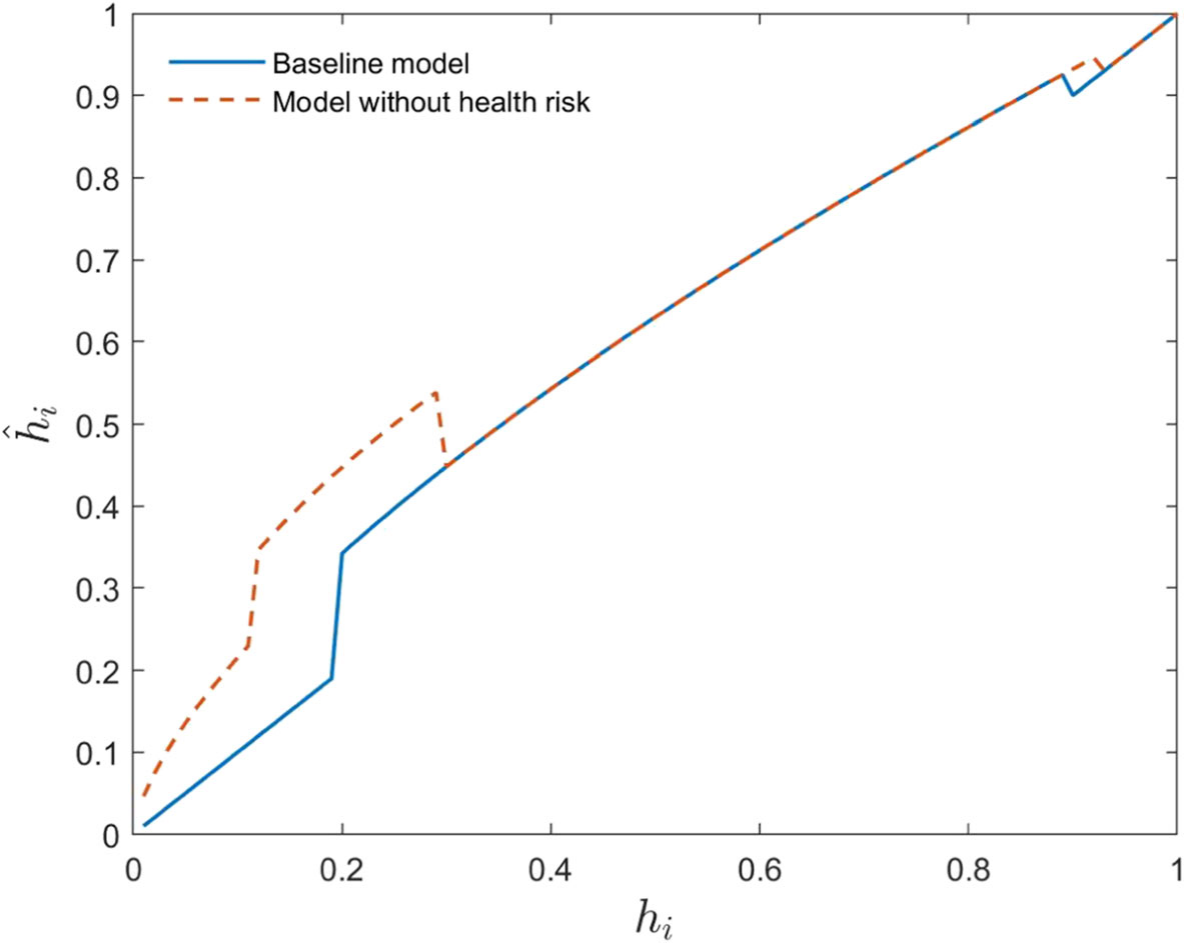
Intergenerational transmission of human capital

The intergenerational transmission of human capital in the steady state is shown in Fig. 2.

**Fig. 3:**
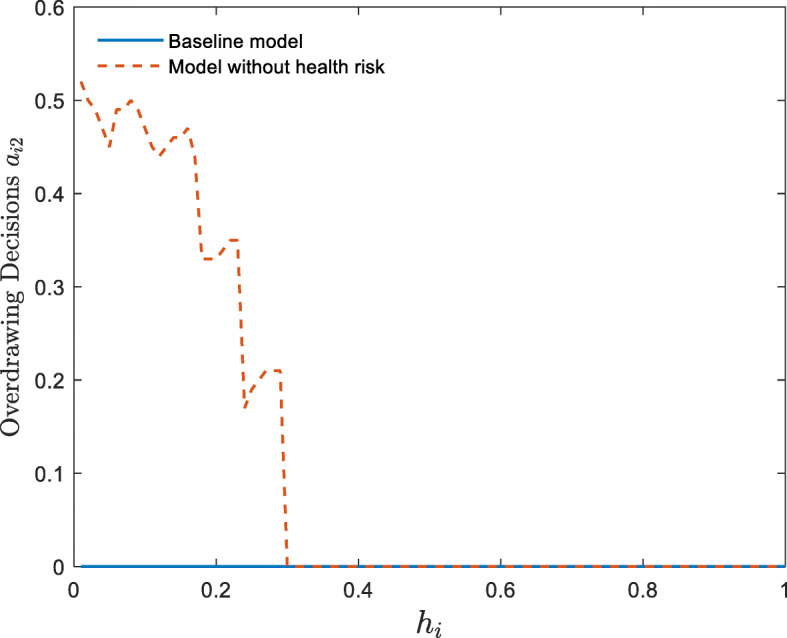
Overdrawing decisions of youth with different levels of human capital (In this study, we use the lattice method to solve the optimal decision of households, so discontinuous changes may occur)

According to the solid blue line in Fig. 2, there are two kinds of households in the steady state: those with low human capital (below 0.19) and those with high human capital (above 0.9). These two kinds of households do not actively invest their assets in education to change their offspring’s human capital, so their offspring have the same human capital as them. However, for those human capital in a range between 0.19 and 0.9, they will invest their funds to improve the human capital of their offspring until their offspring’s human capital is greater than 0.9. Consequently, heterogeneous households will divide into two groups after a certain period of economic development: poor households whose ancestor’s initial human capital is lower than 0.19, and rich households whose ancestor’s initial human capital is higher than 0.19. In the absence of external help, the first group of households will remain in poverty forever. According to the red dotted line in Fig. 2, there is only one type of households in the steady state of the economy without health risks: households with high human capital (above 0.93). Households with initial human capital below 0.93 will continue to invest in their offspring’s education until their offspring’s human capital is greater than 0.93. Consequently, all heterogeneous households achieve prosperity after a certain period of economic development. The absence of health risks is the main reason for this outcome. To achieve human capital growth, households with low human capital choose to overdraw their bodies and earn more income to invest in education when there is no health risk. However, households will think about the future potential healthcare costs of overdrawing their bodies when they have health risks. The difference of overdrawing decisions in these two models is shown in Fig. 3.

**Fig. 4:**
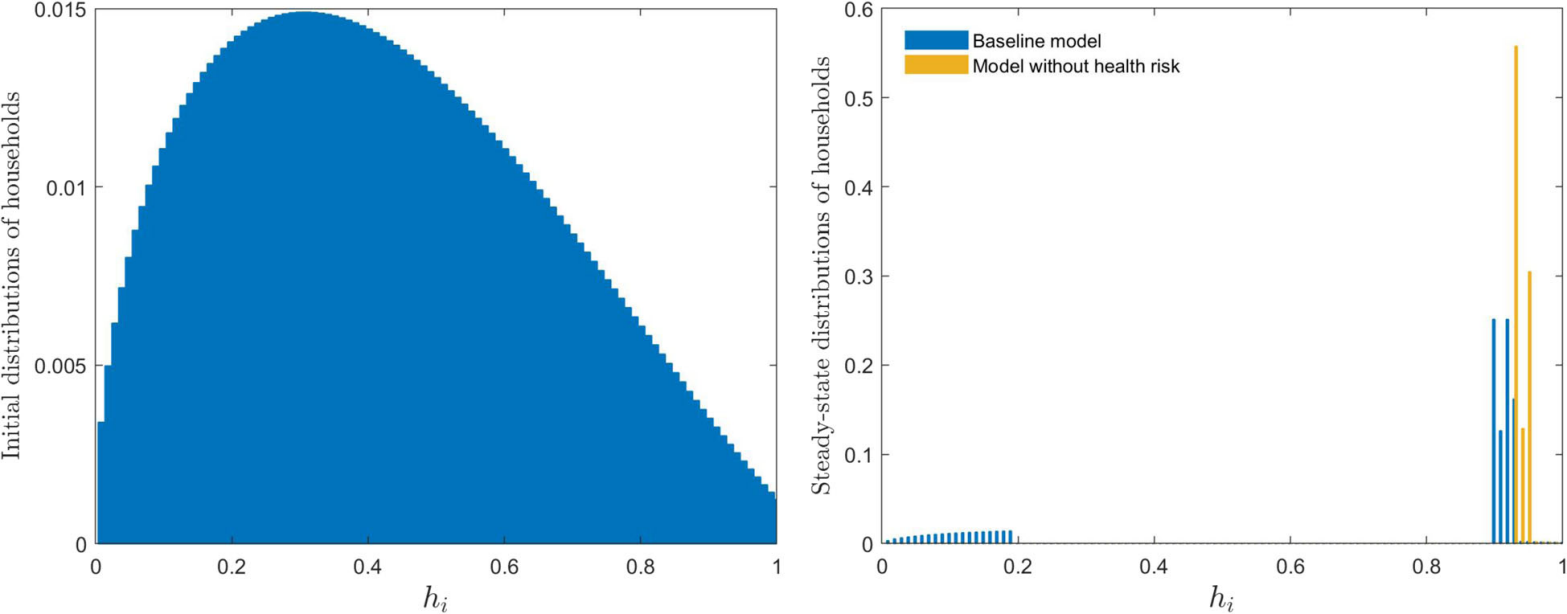
Initial and steady-state distributions of households with different human capital

Figure 3 further support the above explanation that households with low human capital overdraw their bodies to achieve human capital growth when there is no health risk. However, when there is a health risk (baseline model), they do not overdraw their bodies. In line with Fig. 2, Fig. 4 further illustrates the accumulation of heterogeneous households in the future within the framework of this study.

Figure 4 shows that heterogeneous households with an initial log-logistic distribution will accumulate after the long-term development of the economy in this study. In the baseline model, heterogeneous households converge toward two states: the poor state (19.52%) and the rich state (80.48%). However, all heterogeneous households converge toward the rich state in the model without health risks. In addition, households in the model without health risks are more affluent (higher human capital) than those in the baseline model. This is because households in the model without health risks can overdraw their bodies and invest more in education to eventually achieve a higher human capital.

### Results based on health insurance

#### Dynamic paths with health insurance

**Fig. 5:**
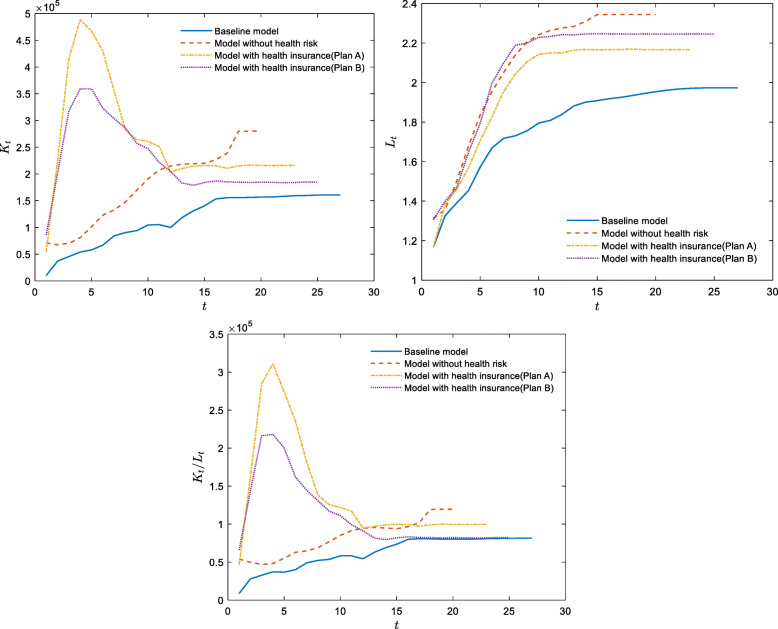
Dynamic paths of the economy with health insurance

Based on the parameters in Table 1, we can solve the utility maximization problem for the household when the health insurance system is included and develop the dynamic paths of total labor, total capital, and capital per capita. Our results are shown in Fig. 5.

In Fig. 5, the yellow and purple dotted lines show the paths of total capital, total labor, and capital per capita under plan A and plan B, respectively. We obtain the effect of the mutual health insurance on the economy by comparing dynamic paths based on the baseline model and those based on the model with health insurance plan. Our results indicate that the health insurance system mitigates the adverse effects of health risks on the economy under plan A or plan B. That is, the total capital, total labor force, and total capital per capita in the steady state based on the health insurance mechanism are higher than that based on the baseline model, but lower than that based on the model without health risks. Therefore, the health insurance mitigates, but does not entirely eliminate, the adverse effects of health risks on the economy. However, the effects of plan A and plan B on the economy are inconsistent. Compared with plan B, plan A leads to higher total capital, lower total labor, and higher capital per capita. The reasons are further analyzed in the next subsection.

#### Steady state and effect of health insurance on poverty alleviation

**Fig. 6:**
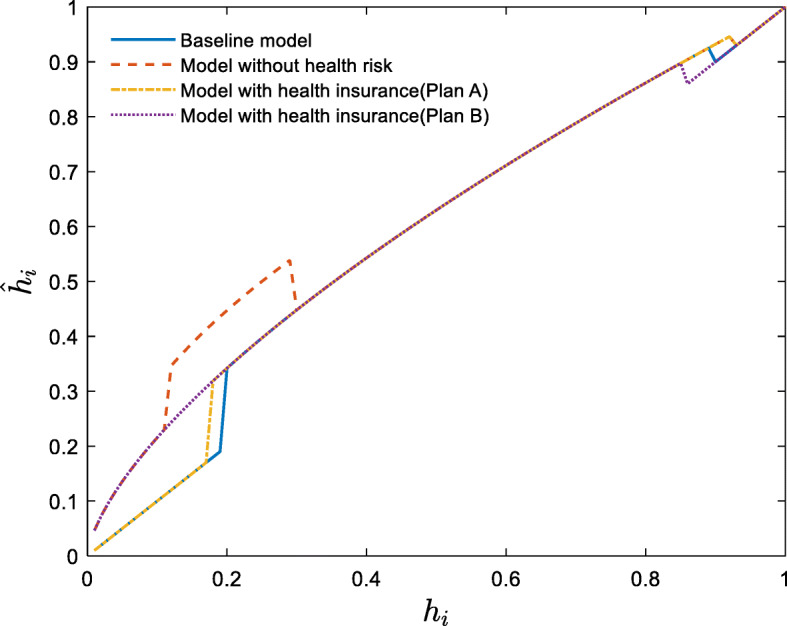
Intergenerational transmission of human capital with health insurance

The intergenerational transmission of human capital in the steady state with health insurance is shown in Fig. 6.

Figure 6 shows the intergenerational transmission of human capital from the father generation to the son generation with the optimal decision in the steady state with health insurance. As illustrated in the Fig. 6, the intergenerational transmission of human capital based on the model with health insurance-plan A (i.e., the yellow dotted line in Fig. 6) is consistent with the structure based on the baseline model (i.e., the blue solid line in Fig. 6). That is, there are two groups of households, the human capitals of some are permanently low and the human capitals of others are gradually increasing until they reach a permanent and invariable high level. However, the model with health insurance-plan B changes the intergenerational transmission rule of human capital in the baseline model, leading to closer match with the model without health risks. Therefore, all households’ human capitals increase gradually until they become permanently and invariably high. Table 2 shows the thresholds for the four models described above.

**Table 2:** Steady-state thresholds

	Poverty threshold	Rich threshold
Model without health risks	–	0.93
Baseline model	0.19	0.9
Model with health insurance model (plan A)	0.17	0.93
Model with health insurance model (plan B)	–	0.86

Figure 6 and Table 2 suggest that the impact of plan A is shown in two ways compared with the baseline model. On the one hand, Plan A decreases the poverty threshold. Adopting plan A lifts households with a human capital of 0.18 and 0.19 out of poverty. On the basis of the data in Fig. 4, this group accounts for 2.76% of the total population and 14.12% of the total poor population. On the other hand, plan A increases the threshold for high human capital in the steady state to 0.93 (i.e., the rich threshold), so households with human capital above 0.93 will no longer seek to increase the human capital of their offspring.

Compared with plan A, the impact of plan B is more significant. Figure 6 and Table 2 show that Plan B lifts all households out of poverty. Since wealthy households share all of the healthcare cost, people caught in the poverty trap obtain human capital growth by overdrawing their bodies to increase their income and education expenditures and, thus, eventually escape poverty. However, the rich households’ wealth decreases owing to the health insurance tax, leading to a decrease in the rich threshold.

Compared with plan A, plan B assumes households in poverty trap do not bear the health insurance tax burden, so the poor households are more willing to overdraw their bodies to escape poverty. However, as non-poor households bear the health insurance tax, they also overdraw their bodies to obtain a break-even point based on their tax contributions, resulting in a larger total labor, a lower unit wage, and a lower capital stock under plan B.

#### Welfare effects of the health insurance system

**Fig. 7:**
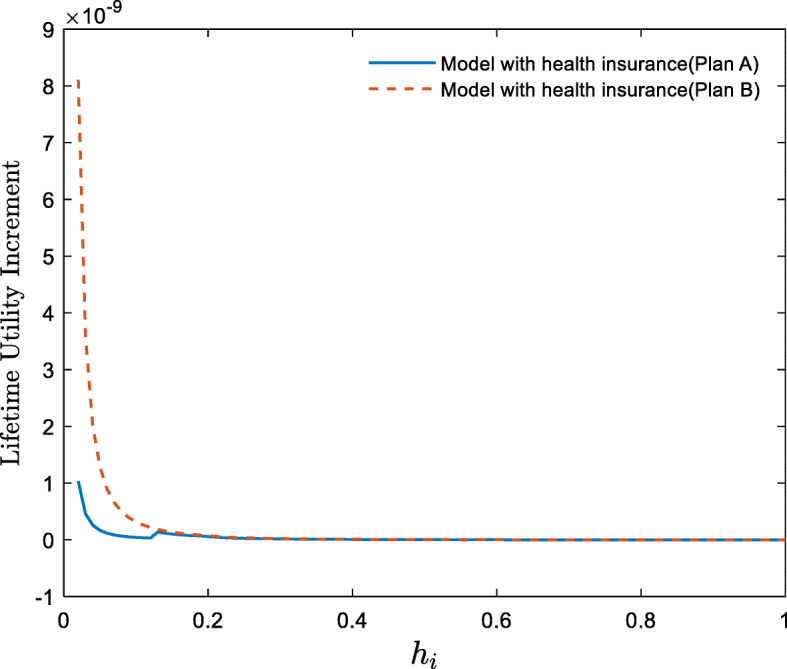
Welfare effects of health insurance systems on households with different levels of human capital (Lifetime utility increment is defined as “lifetime utility with health insurance - lifetime utility in the baseline model”)

According to the results above, health insurance-plan A improves the total economy in the steady state and lifts 14.12% of the poor out of poverty. Meanwhile, health insurance-plan B lifts all households out of poverty but the capital per capita in the steady state remains unchanged. However, does the effect of the health insurance system occur at the cost of some households’ welfare loss? In other words, is the health insurance system a Pareto improvement? This question is analyzed in this subsection, and the results are shown in Fig. 7.

Figure 7 shows that both plan A and plan B increase the total social welfare. Specifically, health insurance-plan A increases the lifetime utilities of all households, health insurance-plan B increases the lifetime utilities of most households.^8^ Therefore, plan A is a Pareto improvement because it increases economic aggregates, lifts some households out of poverty, and increases the lifetime utility of all households; plan B is an effective poverty alleviation plan because it allows all households to increase their offspring’s human capitals and escape poverty through physical overdraft at the cost of minuscule welfare loss for a few households.

## Discussion

### Implications for future research

We contribute to the economics literature in two aspects. First, while previous studies have investigated the impact of family education investment on intergenerational transmission of poverty [12, 31, 39], few studies have considered health risks and endogeneities of income, and then their impacts on economic growth and poverty. Our findings indicate that physical overdraft tends to eliminate poverty but health risk caused by physical overdraft will lead some households fall into the poverty trap. Health insurance arrangement can lift some or even all poor households out of poverty by alleviating the negative impact of health risk on the economy. Therefore, this pioneering study provides a new angle for future scholars to study the relationship among education investment, health and intergenerational transmission of poverty through physical overdraft.

To cope with the health risks caused by physical overdraft and escape from poverty traps, households should buy health insurance because it can transfer part of households’ health risks. Therefore, another contribution of this study is that our proposed health insurance mechanisms can improve the performance of an economy or a region and reduce its poverty level, which will be helpful for policy-makers to make better decision. Ample evidence has shown that insurance mechanism can alleviate poverty. For example, Janzen et al. (2021) found that the introduction of an asset insurance market reduces poverty under two technological assumptions [28]. O’Campo et al. (2004) showed that the increase in unemployment insurance keeps households from experiencing extreme material poverty [37]. Liu (2021) also showed that income protection insurance helps the economy escape from poverty when the insurance coverage is at an optimal level [33]. In line with this, our results indicate that health insurance is a significant determinant of poverty alleviation, and is therefore a major tool available to policy-makers. Our results have important practical implications for economies to escape from poverty trap and reduce income inequality. This is because countries or regions seeking to reduce poverty and income inequalities can adopt similar health insurance mechanisms in this paper. Furthermore, our method has the advantages of achieving or approaching a Pareto improvement and boosting the economic growth.

### Limitations

In this study, we propose an economic development model of endogenous health risks and poverty to investigate the effect of health risks and health insurance on economic growth and poverty. However, we cannot obtain the closed-form solution for our model due to its complexity, such as the effect of physical overdraft decision on health risks and the effect of health risks on households’ budget constraint. Therefore, we set the parameters based on the literature and the actual situation in China to solve the models numerically and analyze it quantitatively. To test whether our results are robust to a different extreme value threshold, we change the value of each parameter in Table 1 and redo the simulation runs for the model with/without health insurance. Our inferences remain unchanged, indicating that we have a credible conclusion.

## Conclusion

This study formulates an economic development model of endogenous health risks and poverty based on a four-period OLG model and investigates the impact of health risk on education investment, intergenerational transmission of poverty and economic growth. In addition, the roles of health insurance in poverty alleviation are analyzed by introducing mutual health insurance mechanisms in this framework. This study is a development upon [12, 31]. Its main contribution is that it endogenizes health risks by assuming that households make the overdrawing decision and then an economic development model of endogenous health risks and poverty is constructed to discuss the effects of health risk and health insurance on the economy and poverty.

Our results show that: Firstly, health risks are the main cause of the poverty trap under the constraint of minimum consumption. Compared with a health-risk-free economy, health risks reduce total capital, total labor, and capital per capita. Based on this result, it can be learnt that the poor will escape the poverty trap through physical overdraft if there is no health risk or the relevant health risks are covered free of charge by health insurance.

In addition, the health insurance mechanism established in this study mitigates, but does not entirely eliminate, the adverse effects of health risks on the economy. Plan A helps a portion of the population below the poverty threshold (14.12% of the poor) escape poverty and is a Pareto improvement. Plan B helps all households below the poverty threshold escape poverty and increases the total social welfare based on the welfare loss of the wealthy. The result means that a health insurance system that covers the poor and has a certain redistributive character can contribute to poverty reduction, and Plan B is such a system.

## Data Availability

All data generated or analyzed during this study are included in this article.

## Footnotes

1 U.S. Census Bureau. “Income and Poverty in the United States: 2020.” https://www.census.gov/library/ publications/2021/demo/p60–273.html

2 There are five theories which attempt to explain the intergenerational transmission of poverty, including the economic resources model (e.g. [7]), the family structure model (e.g. [36]), the correlated disadvantages model (e.g. [25]), the welfare culture model (e.g. [21]), and the social isolation model (e.g. [15]). In the economic resources model, parents must allocate limited resources between current consumption and investment in children’s schooling.

3 A normal family means a family in which the parents are alive, free from domestic violence, pornography, gambling and drugs. Abnormal families and non-family factors are out of the scope of this study. Therefore, we do not consider abnormal families and non-family factors.

4 Many literature has studied the role of health insurance in poverty alleviation, but almost all of them are from an empirical perspective (e.g. [4, 22, 43]. This paper studies the issue from a theoretical perspective.

5 The reason for the setting is to keep $\hat{h_i}$ in (0, 1] and it will ensure the existence of steady state. Obviously, this setting satisfies the general laws among parental human capital, children’s human capital and parental education investment. (1) The higher parental education investment, the higher children’s human capital, for any parent’s human capital. (2) The higher parental human capital, the higher children’s human capital, for any parental education investment. (3) It meets the boundary conditions.

6 Some scholars, such as [50], used a constant elasticity of substitution (CES) function to establish the utility of consumption and physical condition. This study sets a monetary value on physical condition for two reasons. First, the assumption of [50] and ours are not fundamentally different, and the monetary value of physical condition can be derived from the CES function and the monetized power utility function. Second, this study introduces health insurance to analyze its effect on poverty alleviation, which is primarily a monetary compensation for risks. Thus, monetization of physical state will further improve this study’s consistency.

7 http://www.mca.gov.cn/article/sj/tjjb/qgsj/2020/202004.html.

8 Households with utility decrements are not clearly shown in the figure. However, the lifetime utilities of households with human capital of [0.96,1] decrease when adopting health insurance-plan B in the economy. Thus, plan B is not a strict Pareto improvement.
